# Developing a one health data integration framework focused on real-time pathogen surveillance and applied genomic epidemiology

**DOI:** 10.1186/s42522-024-00133-5

**Published:** 2025-02-20

**Authors:** Hanna N. Oltean, Beth Lipton, Allison Black, Kevin Snekvik, Katie Haman, Minden Buswell, Anna E. Baines, Peter M. Rabinowitz, Shannon L. Russell, Sean Shadomy, Ria R. Ghai, Steven Rekant, Scott Lindquist, Janet G. Baseman

**Affiliations:** 1 Washington State Department of Health, 1610 NE 150th St, Shoreline, WA 98155 USA; 2 University of Washington, 1410 NE Campus Parkway, 98195 Seattle, Washington, USA; 3 Washington Animal Disease Diagnostic Laboratory, Washington State University, 1940 Olympia Ave, 99164 Pullman, Washington, USA; 4 Department of Veterinary Microbiology and Pathology, Washington State University, 1845 Ott Rd, Pullman, WA 99163 USA; 5 Washington Department of Fish and Wildlife, Wildlife Program, 1111 Washington St SE, 98501 Olympia, Washington, USA; 6 Washington State Department of Agriculture, 1111 Washington St SE, 98501 Olympia, Washington, USA; 7 British Columbia Center for Disease Control, 655 West 12th Avenue, Vancouver, BC V5Z 4R4 Canada; 8 Centers for Disease Control and Prevention, 1600 Clifton Rd, Atlanta, GA 30333 US; 9 Department of Agriculture Animal and Plant Health Inspection Service, United States, 4700 River Road, 1610 NE 150th St, Riverdale, Shoreline, MD, WA 20737, 418- 5428, 98155 USA

**Keywords:** One health, Epidemiology, Surveillance, Genomics, Informatics

## Abstract

**Background:**

The One Health approach aims to balance and optimize the health of humans, animals, and ecosystems, recognizing that shared health outcomes are interdependent. A One Health approach to disease surveillance, control, and prevention requires infrastructure for coordinating, collecting, integrating, and analyzing data across sectors, incorporating human, animal, and environmental surveillance data, as well as pathogen genomic data. However, unlike data interoperability problems faced within a single organization or sector, data coordination and integration across One Health sectors requires engagement among partners to develop shared goals and capacity at the response level. Successful examples are rare; as such, we sought to develop a framework for local One Health practitioners to utilize in support of such efforts.

**Methods:**

We conducted a systematic scientific and gray literature review to inform development of a One Health data integration framework. We discussed a draft framework with 17 One Health and informatics experts during semi-structured interviews. Approaches to genomic data integration were identified.

**Results:**

In total, 57 records were included in the final study, representing 13 pre-defined frameworks for health systems, One Health, or data integration. These frameworks, included articles, and expert feedback were incorporated into a novel framework for One Health data integration. Two scenarios for genomic data integration were identified in the literature and outlined.

**Conclusions:**

Frameworks currently exist for One Health data integration and separately for general informatics processes; however, their integration and application to real-time disease surveillance raises unique considerations. The framework developed herein considers common challenges of limited resource settings, including lack of informatics support during planning, and the need to move beyond scoping and planning to system development, production, and joint analyses. Several important considerations separate this One Health framework from more generalized informatics frameworks; these include complex partner identification, requirements for engagement and co-development of system scope, complex data governance, and a requirement for joint data analysis, reporting, and interpretation across sectors for success. This framework will support operationalization of data integration at the response level, providing early warning for impending One Health events, promoting identification of novel hypotheses and insights, and allowing for integrated One Health solutions.

**Supplementary Information:**

The online version contains supplementary material available at 10.1186/s42522-024-00133-5.

## Background

Traditional health surveillance systems of humans, animals, and associated ecosystem events or impacts rely on sectorized and independent data systems, analysis platforms, and visualizations [1]. The concept and approach represented by One Health promotes the health and wellbeing of the planet and all living things, pushing our current systems beyond their siloed approaches to coordination and integration. While surveillance systems may vary in their scope, objectives, methods, and platforms, there are general commonalities across surveillance for health events, which include ongoing: (1) sample or data collection, (2) data storage and aggregation, (3) data analysis and interpretation, and (4) dissemination or outcome communication [2, 3]. Moving from a single-sector surveillance system to a One Health surveillance system requires multi-sector coordination at points along this surveillance pathway.

While many organizations, agencies, and authors have called for integration of surveillance data systems across One Health, few examples of systems developed at the sub-national level, where response to health events generally occurs, are available. Those that exist are largely focused on a single condition or health hazard (e.g., West Nile virus, antimicrobial resistance) and are created in response to a known problem [4]. Systems that exist at the national or international scale suffer from reduced timeliness, completeness, and granularity, impeding response at the local level. Challenges currently obstructing systems integration include data dispersion across many domains, heterogeneous data collection methods, lack of semantic interoperability, and complex data governance [5]. Data jurisdiction and organizational mandates differ between sectors, particularly public health, animal health, plant health, and environmental health and food safety [4]. Additionally, informatics capacity varies widely across systems, from paper data collection to complex systems with standardized and automated reporting [5]. Within the United States, state and local governments often have aging data infrastructure and an urgent need for data modernization. Often, funding is vertically allocated with limited or no resources available for cross-sector work, and resources may be scarce even within One Health sectors [4]. At present, there are no state or federal mandates or policies supporting One Health coordination at the local level.

Despite logistical, governance, and financial barriers, the development of integrated One Health data systems has potential to improve prevention and control or management efforts [6]. Specifically, inclusion of laboratory diagnostic data and epidemiological data covering humans, domestic animals, and wildlife alongside environmental data and biodiversity data promises a more holistic picture of One Health events. Coordinating and integrating data across knowledge domains along the surveillance pathway, from collection to dissemination, could provide new insights into One Health-based solutions to address challenges in disease control and prevention at the human-animal-environmental interface, identify novel hypotheses related to health events, and improve early warning for impending health events [7, 8].

In addition to developing integrated One Health data systems, another promising advancement for One Health surveillance is the use of pathogen genomic sequencing and analyses in support of infectious disease surveillance [9]. Pathogen genomic data is host-agnostic, and phylogenetic analysis allows for assessment of transmission dynamics at the human-animal-environment interface. This technology can be applied across bacterial, viral, fungal, and parasitic pathogens. Implementation of integrated genomic surveillance allows for early outbreak detection and improved understanding of pathogen reservoirs, evolution, and modes of transmission, enabling proactive prevention of One Health threats [10]. However, challenges in the development and application of pathogen genomics surveillance systems remain, particularly in government institutions, including laboratory capacity for sequence generation and the capacity to assemble, analyze, and interpret genomic data in real-time [10, 11].

Use of an integrated approach to genomic epidemiology has been most commonly applied in the area of food-borne disease, with the collection of genomic and epidemiological data from human, veterinary, food, and environmental domains in systems such as PulseNet, GenomeTrakr, and the National Center for Biotechnology Information (NCBI) in the United States and the European Food Safety Authority (EFSA) One Health Whole Genome Sequencing (WGS) System in the European Union [12, 13]. The nature of food distribution systems requires national or international coordination; however, data integration alone without joint analysis, interpretation, investigation, or intervention to improve health is not fully representative of a One Health approach [4]. Implementing co-analysis requires building capacity in human, animal, and environmental sectors for producing, sharing, and analyzing sequence data – including hiring and training bioinformaticians [14]. Expansion of a One Health approach to integrated genomic surveillance has not yet been widely extended to zoonotic or vector-borne disease pathogens, despite increasing risk for impact of these pathogens on a global scale and clear multisector benefits for understanding transmission at the local human-animal-environment interface.

Effective genomic epidemiologic and traditional epidemiological analyses require compilation of data and metadata in a systematic way. Integration across domains to develop One Health surveillance systems, including for pathogen genomic data, requires implementation of emerging technologies and database infrastructures, such as application programming interfaces (APIs), artificial intelligence (AI), machine learning (ML), and alternative data systems [5, 11]. Application of these technologies could allow automated data collection from diverse sources and improved cross-domain analytics.

In Washington State government, One Health has been operationalized as a cross-agency collaborative with representatives from One Health institutions meeting quarterly to ensure ongoing collaborative relationships and communication [15]. Additionally, a One Health Surveillance and Data Systems Workgroup meets monthly to improve data sharing, integration, and visualization in support of One Health prevention and response. In 2022, this workgroup began discussing development of an integrated One Health surveillance system for Washington State. However, we lacked a framework for operationalizing One Health data integration at the state level. To better understand the current landscape of One Health frameworks applied to integrate surveillance systems and facilitate co-analysis of multiple data streams, we undertook a study of the existing literature. The overall objective of this work was to identify existing resources and develop a conceptual framework, leveraging concepts from One Health and informatics disciplines, with a focus on pathogen surveillance and genomic data integration, for One Health practitioners to utilize while implementing One Health data integration at the response level.

## Methods

This study used a mixed methods approach, combining a systematic literature review and semi-structured interviews with purposively selected key informants representing One Health, informatics, and genomic epidemiology to understand existing frameworks and examples of cross-sectoral data integration in these domains. The development of a conceptual framework for One Health data integration consisted of four stages: (1) a review of the existing literature to draft an initial framework, (2) key informant interviews including review of the draft framework, (3) synthesis of information and incorporation into design of the framework, and (4) key informant review and revision of the framework for finalization.

### Literature review search strategy and data extraction

We defined the search criteria to focus results related to “One Health” and “Data Systems” or “Data Integration” or “Genomic Data” or “Informatics” or “Digital Health” or “Surveillance System” and searched PubMED and Web of Science using Medical Subject Headings (MeSH) terms. No publication date restrictions were employed. Following the structure provided in the Preferred Reporting Items for Systematic Reviews and Meta-Analyses (PRISMA) Statement, we organized the selection process in three phases: identification, screening, and inclusion [16]. One reviewer (HNO) conducted an initial screening on results, which included peer-reviewed publications, pre-print articles, abstracts, and other reports, for relevance based on title and abstracts. Articles were screened-in if they included mention of One Health and included at least one intervention and/or outcome as outlined in Table 1.

**Table 1:** Scope of the literature review

Population/Problem	Multi-sector (human/animal/environmental health), One Health
**Intervention**	Data systems development, data systems integration, framework for data integration
**Outcome**	Integrated surveillance, integrated data system, genomic data system, One Health informatics, One Health surveillance system, framework

We excluded articles that described an existing surveillance system or process without description of system development or lessons learned, for example, articles:

- About application of genomic analyses to a One Health problem.
- Predominantly focused on describing or evaluating existing surveillance systems, without description of system development.
- Predominantly focused on technological or research advances relevant to One Health (e.g., metagenomic sequencing or viral discovery).

Following this, potentially relevant articles were downloaded and reviewed in full text by one reviewer (HNO). Reference lists of primary articles were searched for additional studies.

Additionally, we searched for relevant gray literature (i.e., websites, reports, protocols, and other documents) on the following agency websites: US Centers for Disease Control and Prevention (CDC), United States Agency for International Development (USAID), United States Department of Agriculture (USDA), Food and Drug Administration (FDA), United States Geological Survey (USGS), United States Fish and Wildlife Service (USFWS), Environmental Protection Agency (EPA), European Centre for Disease Prevention and Control (ECDC), EFSA, One Health Commission, World Health Organization (WHO), Food and Agriculture Organization of the United Nations (FAO), and World Organisation for Animal Health (WOAH). All relevant documents were downloaded in full text and underwent an eligibility assessment for inclusion by one reviewer.

We analyzed all included articles by abstracting the following data elements: title, authors, journal, year, article described or discussed data system development (yes/no), article described or discussed data system integration (yes/no), framework proposed for data integration (yes/no), and framework utilized for data integration (yes/no). Articles were included in this study if the response was ‘yes’ to any of the above questions. In addition, we captured whether articles included discussion of genomic data integration.

### Framework development

Included articles were further reviewed for presence of a framework related to data integration, lessons learned during data system development or integration, or description of framework implementation. We extracted, reviewed, and synthesized these components into the design of a new framework for One Health practitioners to implement One Health data integration, with a focus on pathogen surveillance. A framework implementation guide was also developed to outline specific considerations for each framework step, as well as existing tools or references for each step.

Articles that included discussion of genomic data integration were identified and categorized as: (1) those that discussed genomic data generally; (2) those that gave examples or potential approaches for genomic data integration. Where approaches to genomic data integration were provided, they were summarized, and a figure depicting summarized approaches was developed.

### Key informant interviews

We developed two semi-structured tools to guide discussion for the key informant interviews. One tool focused on identification of existing One Health frameworks, examples of integrated data systems that exemplify this work, and a detailed review of the drafted framework and implementation guide. The second tool focused on identification of frameworks from informatics that may be applied to inform development of a novel framework and considerations for database structures. Purposive sampling was used to select participants, based on area of expertise and involvement in One Health work or data systems and informatics work. We used these key informant interviews to supplement the literature review described above - to identify additional examples of One Health data systems development, One Health data integration, or framework development or application of One Health data integration. A total of 19 individuals were invited via email to participate, 17 of whom agreed and were interviewed during October-November 2023. All interviews were conducted one-on-one in English over video call using one of the semi-structured tools described above. Feedback from these discussions was integrated into framework design, and an updated version of the framework was shared back to key informants for finalization.

## Results

### Literature review

**Fig. 1:**
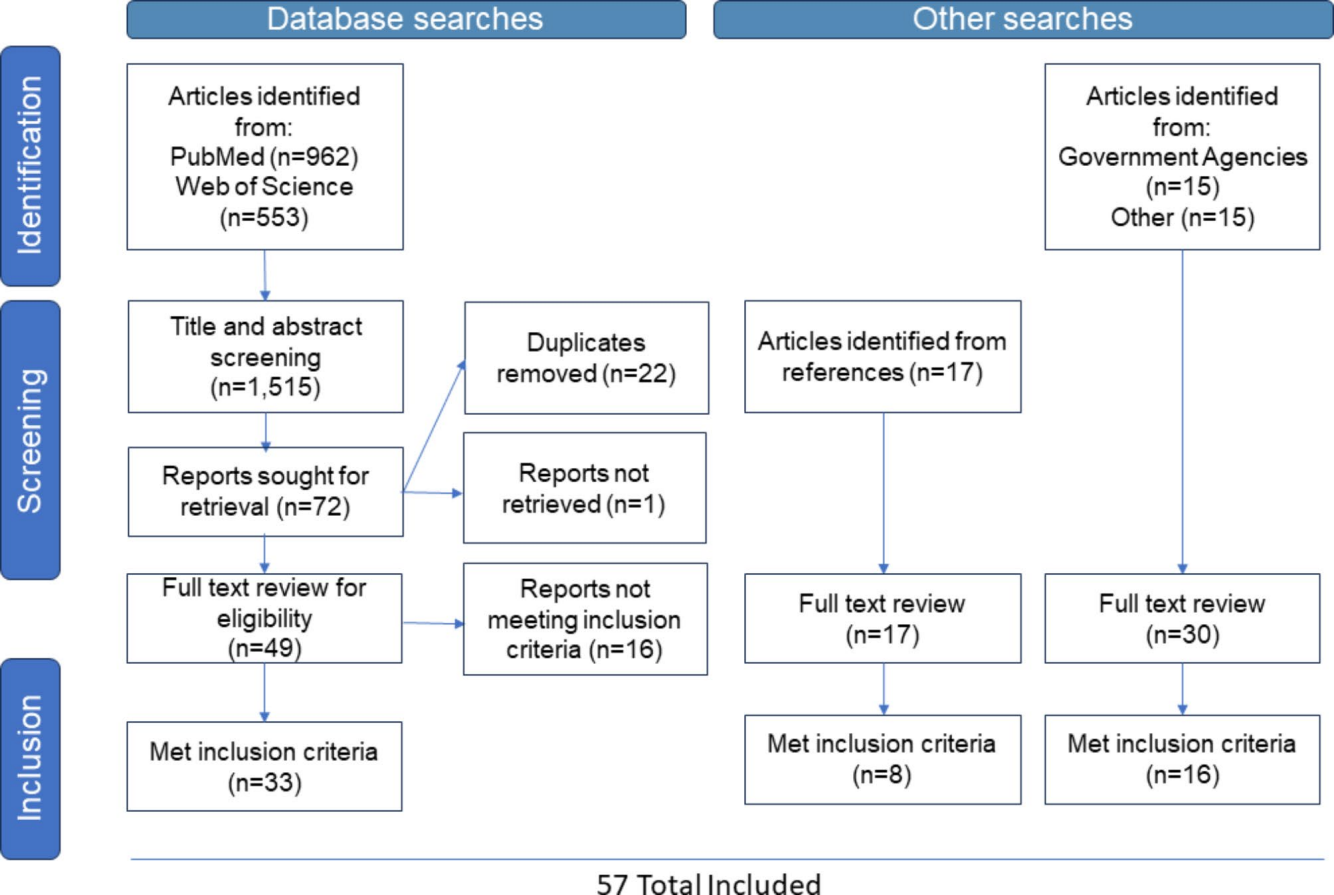
Identification, screening, and inclusion of articles from literature and gray literature searches

The initial literature search identified a total of 1,515 records. Following screening, deduplication, and assessment of inclusion criteria, as well as review of references and gray literature review, 57 records were included in the final study (Fig. 1). From these 57 documents, we identified 2 broad health systems frameworks [17, 18], 3 general One Health frameworks [19–21], and 8 One Health data integration-specific frameworks [2, 4, 6, 22–26] (Table 2). The remaining 44 articles meeting inclusion criteria described integrated data system development [5, 10, 13, 27–45], described general data system development/improvement [8, 11, 46–48], or discussed data system integration [7, 12, 49–63]. Although these articles did not define specific frameworks, lessons learned during data system development or integration and best practices in data integration were included in our final framework and implementation guide, which outlines specific questions and considerations for each framework step, as well as existing tools or references that may be useful (Supplementary Material).

**Table 2:** Health systems, general one health, and one health data integration frameworks identified during literature review

Name/Title	Reference	Description	Factors implemented into study framework
**Health Systems Frameworks**			
WHO Ten steps to systems thinking in the health system	18	Tool for applying systems thinking to health systems	Considered steps in development of final framework, notably including funding and a consideration of unexpected outcomes, as well as baseline and post-evaluation.
UNECE Generic Statistical Business Process Model	17	Generic process model for production of official statistics	Integrated steps from “specify needs” into system scoping.
**General One Health Frameworks**			
GOHF (Generalizable One Health Framework – CDC)	19	Generalizable One Health framework for the control of zoonotic disease	Ensured activities represented in the generalized framework were covered in developed framework.
Overarching One Health conceptual framework	20	Implementation cycle to inform One Health tool use	Integrated steps of cycle into overall framework structure.
OH-SMART	21	Multi-sectoral health system analysis and process improvement toolkit	Integrated OH-SMART steps into the planning/pre-funding steps of the final framework.
**One Health Data Integration-Specific Frameworks**			
Matrix Integrate-OHSS	2; https://ejp-matrix.eu/	A framework to develop a One Health surveillance System from an existing system	Integrated steps into overall framework structure, utilized multiple resources to develop implementation guide.
One Digital Health	22, 70	A framework for future health ecosystems, joining the concepts of Digital Health and One Health	Integrated aspects of data standardization and interoperability, consideration of novel data sources
Socio-technical framework to develop common stakeholder vision for surveillance	23	A framework to help stakeholders develop a common vision of their desired surveillance system and forge the innovation pathway toward it	Consideration of strengthening existing surveillance capacities as part of the framework for integration. Framework steps 1–4 integrated in the final framework.
Standardized framework for data integration	24	Outlines essential data elements and a consistent reporting template, including mapping to SNOMED codes	Integrated into step 6 “Outline data design and user requirements.”
A conceptual framework for organization of collaboration in a One Health surveillance system	4	Outlines organization factors conducive to sustainable collaboration, as well as aspects of collaboration supporting One Health surveillance systems	Integrated in the partner identification (1) and funding plan (5) steps of the framework.
A Tripartite Guide to Addressing Zoonotic Diseases in Countries Sect. 5.2.2	6	Outlines elements for establishing a comprehensive, coordinated system for surveillance and information sharing	Integrated outlined elements into overall framework.
Tripartite Surveillance and Information Sharing Operational Tool	25	Tool for establishing or strengthening a One Health multi-sectoral coordinated surveillance and information sharing (SIS) system for zoonotic diseases	Integrated outlined elements into overall framework, utilized multiple resources to develop implementation guide.
OHHLEP One Health surveillance system development framework	26	Outlines 6 steps to overcome barriers and optimize an integrated One Health Surveillance system	Integrated outlined elements into overall framework.

### One health data integration framework

**Fig. 2:**
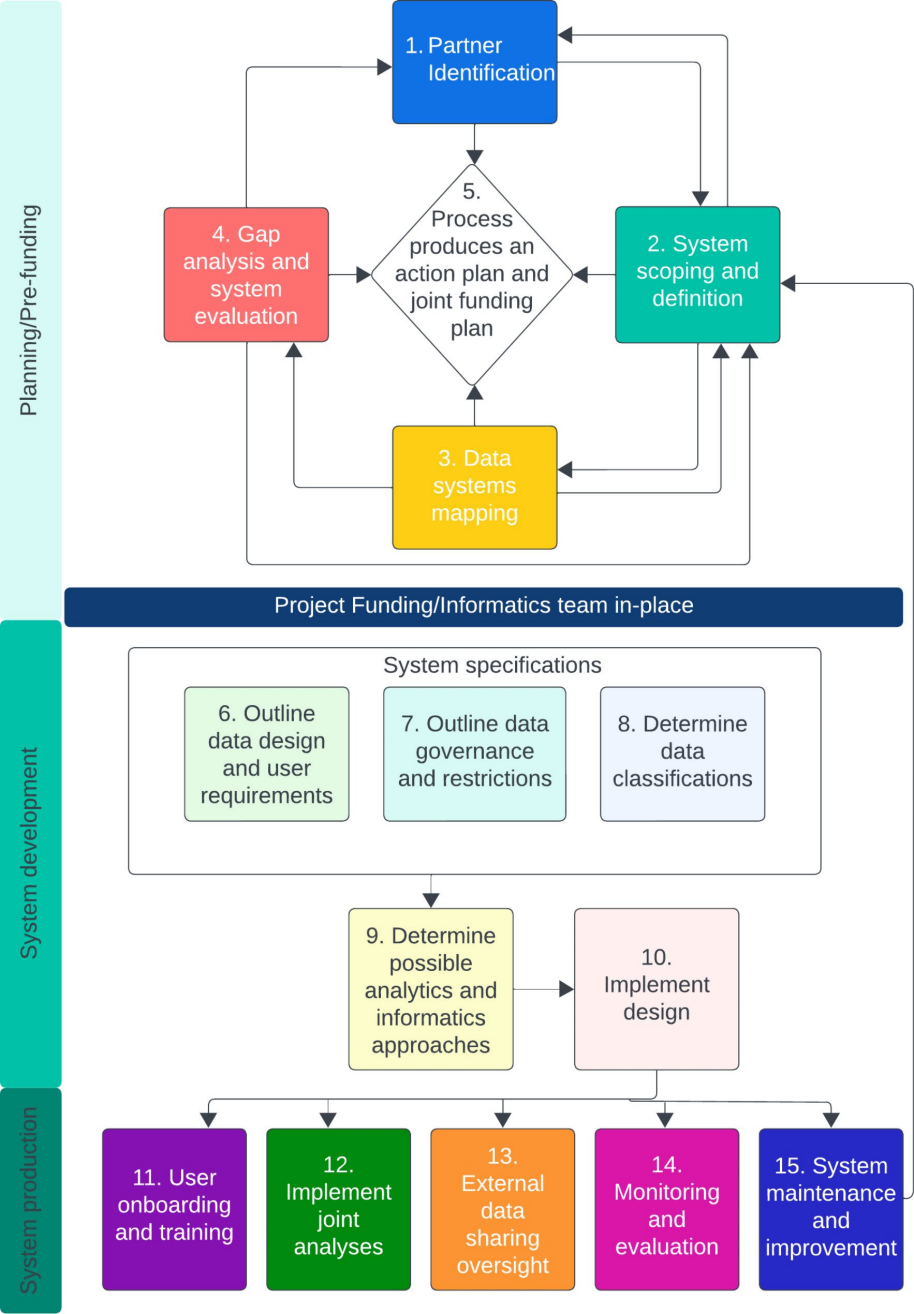
A One Health Systems Framework for Data Integration. First, a workgroup is formed, considering participants from sectors that collect, analyze, and have governance over relevant data, as well as from different disciplines, the research community, public-private partnership, or community partners. This workgroup scopes the system to clearly define the purpose and outputs. Based on the specified scope, a data mapping process is performed to understand what relevant data is available and whether the data timeliness, completeness, granularity, and quality support the scope. Current data structures, access, and connections are defined, and system capabilities (such as data export formats or capacity to send or receive standardized messaging) are outlined. Partner inclusion may be assessed throughout the process of data mapping, and system scope may need alterations dependent on available data. Based on the differences in the desired system and the mapped current system, gaps in data, data linkage, and data access are identified. Recommendations for improved data capture or sector-specific data systems to support a future integrated data system may be needed. An action and funding plan should be jointly developed throughout this process. A One Health data integration informatics team should be in-place prior to system development. In the system development phase, system specifications are outlined, including the data design, user requirements, data governance, and data security levels. Based on these system specifications, database structure options are considered, prioritizing modern data connections, limiting manual data manipulation, and future system needs for flexibility. The selected approach is used to identify potential system options. Once a system is in-place, the production phase should include user onboarding and training, system documentation, implementation of joint analyses, external data sharing oversight, ongoing monitoring and evaluation, and a plan for system maintenance and improvement. This last step may include revisiting system scope to add future capacity. See the framework implementation guide for additional detail

Common and unique elements of the above-identified frameworks were outlined; combined with the authors’ own experiences, these were integrated into a draft data integration framework (Table 2). One Health expert interviewees included 12 representatives of state agencies of public health, agriculture, and fish and wildlife; federal agencies of public health and agriculture; and university representatives. Five informatics expert interviewees represented state and federal public health agencies and university representatives. The final framework and implementation guide were developed following feedback from these interviews and final revision by the interviewees (Fig. 2, Supplementary Material).

Unique perspectives captured in the One Health expert interviews that altered the framework included the cyclical nature of the planning stage where system scoping, data mapping, and gap analysis re-inform partner identification, and each step informs an action and funding plan. Consideration of data governance and external data sharing were highlighted as critical, and additional considerations and resources on these topics were added to the implementation guide. A step for monitoring and evaluation was also added to capture the need for iterative evaluation and improvement of an integrated system. A requirement for system flexibility and future re-scoping and improvements was highlighted. Additionally, a framework not previously identified through the literature or gray literature reviews was identified through expert interview: the Integrated Disease Surveillance and Response (IDSR) Sect. 9: Electronic Integrated Disease Surveillance and Response [64]. This framework was reviewed and incorporated into the implementation guide.

Informatics expert interviews identified the subject-agnostic problems of data integration and interoperability, as well as commonalities of the framework’s planning stage to the work normally performed during business process mapping efforts. However, these efforts generally occur within the funded scope of an existing project at a single agency/institution. Within this One Health framework, funds are generally unavailable in the planning stages, often prohibiting informatics support, and the scope is not clearly defined at the outset. To ensure that adequate business process mapping is performed, working groups will likely need to revisit the scope, data mapping, and gap analysis after onboarding informaticians, prior to system specification. Emphasis was placed on clear identification of the problem to be solved and outlining the specific outputs of an integrated system. One key example of system scoping was an approach where partners work through an exercise to agree on 20 questions the system should be able to answer, including enough specifics (such as time period and location), to inform data design [65]. The requirements inherently outlined in these questions can then be used to specify the system scope and work toward system specifications. Emphasis was also placed on data collection and integration even in advance of standardization within this large of an integration effort to avoid getting stuck in system specification, as well as starting from raw data and including data transformation code within the system (e.g., using Structured Query Language (SQL)). Finally, with respect to flexibility for future system changes, changing infrastructure and technology should be considered in addition to changing surveillance priorities.

### One health genomic data integration

Genomic data integration was addressed by 10/57 articles (18%). Of these, 3 discussed the growth in genomic data production and analysis, the importance of considering genomic data when conducting integration, or the need for Standard Operating Procedures for laboratory and bioinformatic efforts across One Health sectors [2, 5, 26]. Seven articles outlined examples or potential approaches for genomic data integration [8, 10, 11, 13, 28, 35, 38] (Table 3). Across these articles, common themes included pairing sequence data with a critical set of metadata according to a set of standards, standardization in quality checks and bioinformatics pipelines, and analysis across One Health sectors. Additional best practices included controlled data access to allow restricted and public views (i.e., data sharing and access principles), a process for data updates and corrections, connections of distributed databases through APIs, reproducible analyses, expanding the technical workforce across sectors, and open data sharing. In particular, WHO’s “Global genomic surveillance strategy for pathogens with pandemic and epidemic potential, 2022–2023” calls for both leveraging genomics across One Health sectors and making the use of genomics routine in surveillance practice and disease prevention, preparedness, readiness, and response [8].

**Table 3:** Potential approaches for one health genomic data integration

Reference	Overview	Approach
28	Describes a shared secure surveillance platform between human and veterinary medicine in Switzerland	• Includes human, animal, environmental, and food isolates • Includes sequence data and associated metadata • Features controlled data access • Allow complex dynamic queries • Features dashboards • Automated data sharing with international repositories • Incoming data are quality- checked, curated, standardized where needed and processed/annotated with dedicated bioinformatics pipelines
10	Describes a shared platform for multisectoral data collection and bioinformatic analysis in Italy	• Includes isolates from food, environment, human and non-human and associated metadata • Bioinformatics tools sharing a common workflow system • Process for quality control
13	Outlines best practices for One Health contributions to open access databases	• Store the sequence with metadata • Inclusion of specimens from human, animals, food, and environment • Thresholds for QC • Contact information for submitters • Process for updating data, responding to requests, and correcting submissions
35	Describes a federated ecosystem as a possible solution for sharing genomic data	• Encourages use of federated databases • Connections of distributed databases through APIs
38	Describes a One Health system for hepatitis E virus surveillance in Europe	• Inclusion of human, animal, food, and environmental samples • Secure online environment • All sequence data with a restricted set of associated metadata become publicly available at a time specified by the data provider
11	Describes 10 recommendations for an informatic ecosystem to support pathogen genomic analysis in public health agencies	• Consistent data model (pairing sequence data with metadata) • Strengthen APIs to automate querying and analysis • Data management and stewardship • Bioinformatics pipelines open-source and accessible • Develop modular pipelines for data visualization and exploration • Improve the reproducibility of bioinformatics analysis • Utilize cloud computing • Expand the technical workforce • Improve the integration of genomic epidemiology with traditional epidemiology • Best practices to support open data sharing
8	Describes 5 objectives for genomic surveillance strengthening	• Improve access to tools for better geographic representation • Strengthen the workforce to deliver at speed, scale, and quality • Enhance data sharing and utility for public health decision-making and action • Maximize connectivity • Maintain a readiness posture for emergencies

**Fig. 3:**
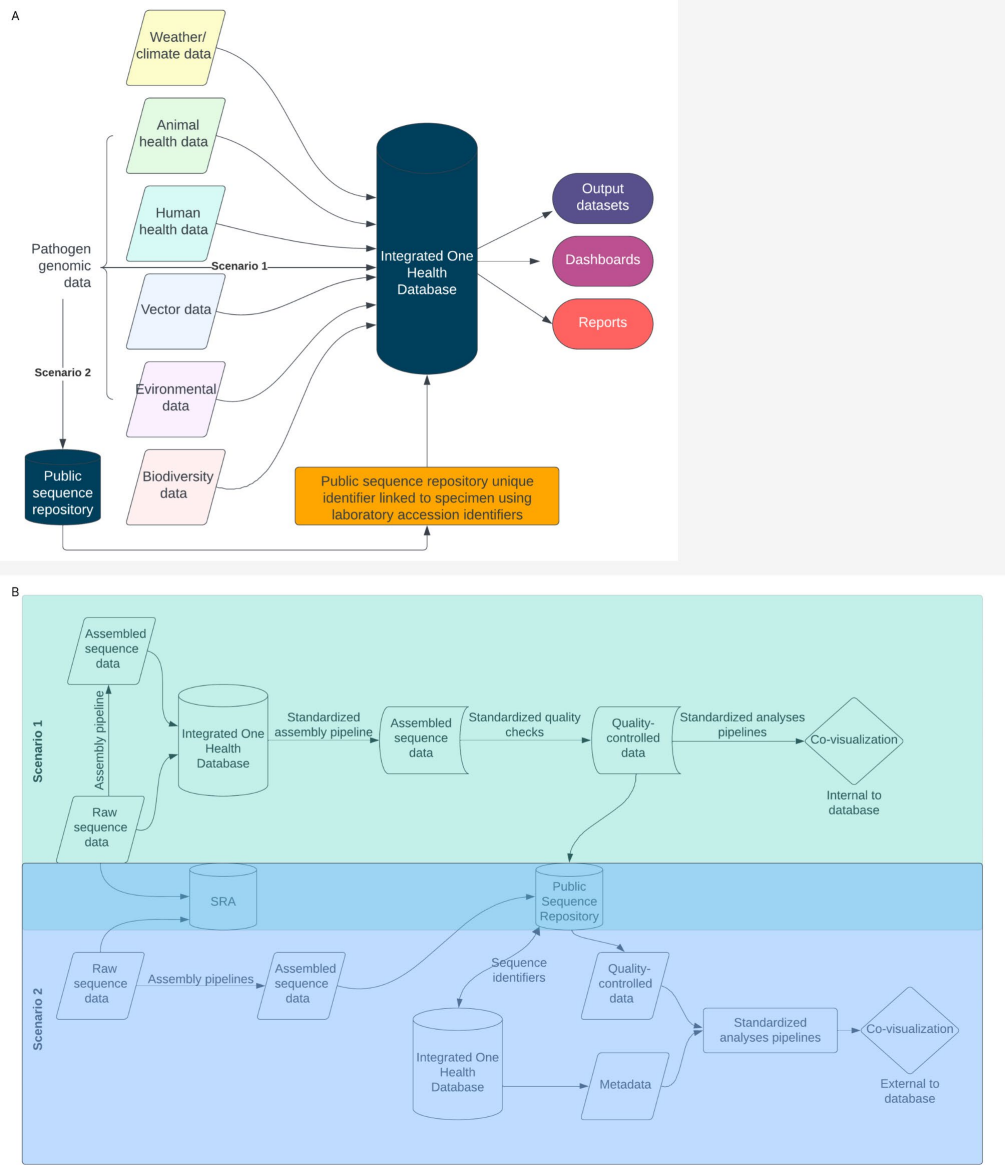
Model one health database and scenarios for genomic data storage (**A**) Model One Health Integrated Database, (**B**) Elaboration of scenarios for genomic data storage and analysis. Scenario 1: sequence data is stored within the integrated One Health database, which contains standardized assembly pipelines and quality checks as well as analysis pipelines and visualization tools. Scenario 2: sequence data is stored external to the integrated One Health database and linked through public sequence repository identifiers. To conduct analyses, sequence data is extracted from a public repository, combined with extracted data from the integrated One Health database, and analyzed using standardized pipelines. Analysis and visualization occurs external to the integrated One Health database

Overall, there were two proposed structures that could be applied to One Health genomic epidemiologic data storage and analysis: (1) the integrated database is configured to store sequence data (either raw sequencing read data or both raw and assembled data) alongside metadata, with a process for standardized upload to public repositories; or (2) the integrated database stores only the public repository sequence identifiers alongside the metadata (Fig. 3). All implemented examples in the literature represent scenario one. In this scenario, platforms allow for local analysis and visualization with controlled access but require substantial infrastructure and support [10, 28, 38]. In the second scenario, sequence data from public repositories would need to be extracted and combined with metadata prior to analyses using tools external to the common platform [11]. In either scenario, the integrated genomic data should be generated with validated assays, assembled using standardized assembly pipelines that include quality checks across hosts, and integrated with other One Health data prior to joint visualization and analysis, with interpretations communicated across sectors.

## Discussion

We undertook a literature search to inform development of a conceptual framework for One Health data integration, focusing on pathogen surveillance and genomic data. Although existing frameworks for One Health data integration were identified, none reflected the full scope of undertaking this work at the local or state level, instead focusing mainly on planning stages. Similarly, none were identified as implemented for integration at this level. We sought to develop a framework that better captures elements required for start-to-finish implementation of integrated One Health surveillance at the response level. To do so, we combined the results of the literature review, the authors’ experience in Washington’s One Health Surveillance and Data Systems Workgroup, and expert interviews that included informaticians and One Health experts from inside and outside this workgroup. In particular, our overlay of key One Health considerations with generalized informatics frameworks is a novel approach to framework development in this topic area. We believe this framework can be utilized by other jurisdictions seeking to undertake data integration at the response level.

We identified eight previously published One Health data integration-specific frameworks. Of these, two provided substantial resources and were the most informative to the proposed framework: the Matrix Integrate One Health Surveillance System (OHSS), which provided a step-by-step guide to creating a One Health surveillance system from existing surveillance programs (https://ejp-matrix.eu/overview/)^2^, and the Tripartite Zoonoses Guide Surveillance and Information Sharing Operational Tool (SIS OT) [25]. In particular, the data mapping tool from OHSS provided useful considerations to assist jurisdictions in conducting the data mapping step of the process. This tool and other relevant tools are referenced throughout our framework implementation guide (Supplementary Material). Of these eight frameworks, only three considered the identification of funding or other resources as part of the framework [4, 6, 25]. None except SIS OT adopted feedback loops, instead proceeding in a stepwise fashion from start to finish. Most considered the steps reflected in our planning/pre-funding phase but did not consider steps following resource allocation. Developing these continuing steps of the framework gives jurisdictions a path beyond envisioning this system to move toward implementation. In addition to working toward data integration, One Health partners will need to consider how surveillance efforts supported by the data integration platform will support prevention and response. In parallel to data integration processes, jurisdictions should develop an integrated plan for conditions under surveillance, including how integrated data visualization and analyses will be utilized in support of prevention and response activities. This plan can help to inform the implementation of framework steps, including data design, analytics approaches, and implementation of joint analyses.

During the expert interviews, we discussed how this framework differed from one that would be commonly applied to general issues of data interoperability and systems design, such as a general data management framework or system development lifecycle (SDLC) model. A general data management framework may consider elements such as data governance, data quality, data integration, data security, data privacy, data retention, data architecture, and data analytics. An SDLC encompasses planning, analysis, design, development, integration and testing, implementation, and maintenance. Although these generalized frameworks were reviewed to ensure consideration of all aspects across our framework, placing this framework within the One Health context requires several important adaptations. First, partner identification is complexified by consideration of professionals in various sectors across human, animal, and environmental health who may include collectors, users, or interpreters of relevant data. The system scoping and definition is not an obvious or clearly defined problem as may often be the case within a single-sector data integration problem, instead requiring engagement and co-design across sectors. Data systems mapping spans agencies and institutions and connections between sectors may not be apparent. Each of these steps may result in feedback loops to the previous steps. Although the planning stage may reflect the work commonly performed during business process analysis, this step is performed in advance of funding allocation and likely does not include a trained informatician or analyst to support the effort. Data governance conversations will be critical and will likely define parts of the system scope. Government agency information technology (IT) requirements will limit possible informatics approaches. Finally, the requirement for planning for joint analysis, reporting, and interpretation across sectors is unique to the One Health model.

Genomic data integration was considered in one previous framework: the One Health High Level Expert Panel (OHHLEP) One Health surveillance system development framework acknowledges that design aspects should accommodate technological advances, such as WGS. When discussing specific disciplines for implementation, laboratories are identified as an area where integration can occur both at the testing and analysis levels, including sequencing and bioinformatic, genomic, phylogenetic, and phenotypic analyses [26]. In addition, the One Health Surveillance Codex, which includes Integrate-OHSS, also includes a “Sequencing for Surveillance Handbook” [2, 66]. This handbook is not referenced within Integrate-OHSS but is a separate tool. Consideration of different data types, including genomic data, is vital to system design and successful implementation, even in settings where this capacity is not yet available. Failure to consider and include genomic data or data identifiers in a One Health system application may result in rapid obsolescence, as infectious disease surveillance is increasingly reliant on genomic epidemiology to support surveillance and investigation, as well as hypothesis generation.

We outline two scenarios identified in the literature for inclusion of genomic data in planning for an integrated One Health data system. In scenario 1, either raw or raw and assembled sequence data are included internal to the system, in scenario 2, sequence identifiers are included as linkages without storage of the full sequence data. All implemented examples in the literature represented scenario 1. The potential benefits of this scenario were identified as internal standardization of assembly and quality checks, visualization of integrated data within a controlled-access system, and improved opportunities for shared analysis resources and joint analyses. However, substantial storage capacity to maintain full sequence data and infrastructure development for storage and computation are required. Computing resources available to the developed infrastructure may limit the types of analyses that could be performed. A potential modification of this scenario is the storage of assembled sequences only and the use of the Sequence Read Archive (SRA) for raw data storage; however, this removes the possibility of standardized assembly pipelines within the system which was outlined as a major benefit.

Scenario 2 potentially allows for more flexibility in selection of analysis and visualization tools and removes the storage burden of sequence data. However, this scenario requires establishing a process for linking sample identifiers (such as laboratory accession numbers) to repository unique identifiers to ensure linkage is maintained. In Washington State, scenario 2 has been implemented with respect to linked human genomic and epidemiologic data, and we foresee this as a scalable and preferred option for most databases, especially those developed in low-resource settings. Standardized quality checks are already in place in public repositories, and this option provides flexibility in case the amount of data exceeds storage capacity, as well as flexibility in the selection of analysis and visualization tools. In many cases, access to raw sequence data may not be available, and data within public repositories may represent a wider capture of sequence data. However, one limitation of this scenario is the inability to perform standardized assembly, requiring additional coordination at the laboratory and bioinformatics levels. Integration at the laboratory level provides opportunities for increased efficiency and cost-effectiveness, while removing barriers to data integration.

One key requirement of integrated genomic epidemiologic analysis of emergent surveillance data is the availability of a platform for joint analysis and visualization within a controlled-access system. Indeed, this requirement appeared to drive the selection of scenario 1 in most if not all instances identified. Alternatives to developing additional systems for visualization and analysis while allowing for controlled access include shared analysis files for visualization with Nextstrain Auspice (https://auspice.us/), developing a Nextstrain group (https://nextstrain.org/groups/), or using an alternative visualization platform with controlled access, such as Data Flow and MicroReact [67, 68]. Each of these alternatives come with their own challenges for process development that must be considered.

This review is subject to several limitations. The search strategy for inclusion of articles focused on those containing reference to One Health; therefore, this review does not provide an exhaustive overview of cross-sector data integration frameworks. A single author performed article screening and review for inclusion; review by multiple authors may have led to identification, inclusion, or exclusion of additional articles that may be relevant to this study. The focus of this review targeted pathogen surveillance and genomic data integration – there are also many health conditions unrelated to pathogens that require a One Health approach. Although we did not specifically address these other health conditions and the need for novel data sources such as from the social or behavioral sciences, this framework may likely be extended for application to non-infectious condition sources. Likewise, our work focused on the development of an integrated system from existing siloed systems; an ideal surveillance system likely includes elements that may not yet exist at the local level such as animal health syndromic surveillance, citizen science reporting across sectors, and robust veterinary laboratory data reporting from commercial laboratories. Development of new primary systems was not included outside of consideration of areas for improved primary data capture or data systems.

Many of the identified frameworks included overlapping elements; indeed, although we re-conceptualized the planning stage of our framework as cyclical instead of stepwise, these elements were reflected across other frameworks. In addition to the cyclical nature of the planning stage, the novelty of our framework is in the expansion to system development and production, providing a framework for the path forward to implementation, and in the overlay of informatics frameworks and concepts. We emphasize the importance of considering multi-level data, including plans for pathogen genomic data early in the process, to ensure a holistic One Health surveillance approach that recognizes the increasing importance of genomic epidemiology in infectious disease surveillance methods. The development of this framework makes it clear that both a coordinating workgroup and a technical workforce with expertise in informatics are required to support the development of One Health systems. The lack of such available expertise devoted to One Health work may partially explain the dearth of integrated systems at the response level. Jurisdictions seeking to develop One Health systems in the absence of engaged support from a coordinating workgroup and workforce could potentially make progress through smaller-scale data collection and integration in advance of system standardization. Such work may allow for additional advocacy and funding for larger multi-agency One Health projects.

Government data systems are often outdated and do not often make use of technologies like artificial intelligence and machine learning. These technologies hold great future promise for overcoming challenges such as lack of semantic interoperability, data mapping, and data integration [5]. Modern data architecture should be considered key in the sustainable development of integrated One Health data systems. To ensure not only the development of integrated systems but also their sustainability, this framework outlines developing common goals, strong governance, and routine coordination and communication [6]. In addition, policy or legislation change is considered at multiple steps, to improve the landscape for data collection, data sharing, and process support. In Washington State, this work was exemplified during recent efforts to improve data sharing between the Washington State Department of Agriculture and the Washington State Department of Health; in addition to co-creation of a new process for data collection and cross-reporting, the Washington Administrative Code was updated to require reporting of animal diseases of public health concern.

## Conclusions

Conducting real-time surveillance and response using the One Health approach requires a range of expertise both across One Health sectors and across disciplines, such as epidemiology, veterinary medicine, genomics, bioinformatics, informatics, and laboratory sciences. Benefits are gained not just from integrating data but from conducting joint analyses, bringing together a sufficient range of expertise to improve early detection and response [69]. This One Health data integration framework will help jurisdictions operationalize this work at the response level, moving past envisioning a system to allow implementation of systems development, leading to joint analyses and response. As such, it can serve as a model for implementation at the national level as well. The framework’s focus on real-time pathogen surveillance and genomic data integration reinforces, modernizes, and expands our joint ability to prevent and control disease for the health of humans, animals, and the environment we share.

## Electronic supplementary material

Below is the link to the electronic supplementary material.

Supplementary Material 1

## Data Availability

The datasets used during the current study are available from the corresponding author on reasonable request.

## Abbreviations

AI: Artificial intelligence
API: Application programming interface
CDC: US Centers for Disease Control and Prevention
ECDC: European Centre for Disease Prevention and Control
EFSA: The European Food Safety Authority
EPA: US Environmental Protection Agency
FAO: Food and Agriculture Organization of the United Nations
FDA: US Food and Drug Administration
MeSH: Medical Subject Headings
ML: Machine learning
OHSS: Matrix Integrate One Health Surveillance System
SDLC: System development lifecycle
SRA: Sequence Read Archive
SIS OT: Tripartite Zoonoses Guide Surveillance and Information Sharing Operational Tool
USAID: US Agency for International Development
USDA: US Department of Agriculture
USFWS: US Department of Fish and Wildlife Services
USGS: US Geological Survey
WHO: World Health Organization
WOAH: World Organisation for Animal Health

## References

[1] Oltean HN. Enhancing public health surveillance: integrating genomic and epidemiologic data to inform public health action and one health progress. University of Washington; 2023.

[2] Filter M, et al. One Health Surveillance Codex: promoting the adoption of one health solutions within and across European countries. One Health. 2021;12:100233. 33786360 10.1016/j.onehlt.2021.100233 PMC7994538

[3] Armstrong G et al., *Updated Guidelines for Evaluating Public Health Surveillance Systems Recommendations from the Guidelines Working Group. 1–35 (2001).* 18634202

[4] Marion Bordier TU-A, Aurélie Binot P, Hendrikx FL. Goutard. Characteristics of one health surveillance systems: a systematic literature review. Prev Vet Med. 2020;181:104560. 30528937 10.1016/j.prevetmed.2018.10.005

[5] Ho CWL. Operationalizing One Health as one Digital Health through a global Framework that emphasizes Fair and Equitable sharing of benefits from the Use of Artificial Intelligence and Related Digital technologies. Front Public Health 10(2022). 10.3389/fpubh.2022.768977 PMC9110679 35592084

[6] FAO O. WHO. Taking a Multisectoral, One Health Approach: A Tripartite Guide to Addressing Zoonotic Diseases in Countries. (2019).

[7] Aenishaenslin C, et al. Evaluating the Integration of One Health in Surveillance Systems for Antimicrobial Use and Resistance: a conceptual Framework. Front Vet Sci. 2021;8:611931. 33842569 10.3389/fvets.2021.611931 PMC8024545

[8] WHO. Global genomic surveillance strategy for pathogens with pandemic and epidemic potential, 2022–2032. (2022). 10.2471/BLT.22.288220 PMC8958828 35386562

[9] Urban L et al. Real-time genomics for one health. Mol Syst Biol, e11686 (2023). 10.15252/msb.202311686 PMC10407731 37325891

[10] Knijn A, et al. IRIDA-ARIES Genomics, a key player in the One Health surveillance of diseases caused by infectious agents in Italy. Front Public Health. 2023;11:1151568. 37361153 10.3389/fpubh.2023.1151568 PMC10289303

[11] Black A, MacCannell DR, Sibley TR, Bedford T. Ten recommendations for supporting open pathogen genomic analysis in public health. Nat Med. 2020;26:832–41. 32528156 10.1038/s41591-020-0935-z PMC7363500

[12] Aarestrup FM, Bonten M, Koopmans M. Pandemics- one health preparedness for the next. Lancet Reg Health Eur. 2021;9:100210. 34642673 10.1016/j.lanepe.2021.100210 PMC8495373

[13] Timme RE, et al. Optimizing open data to support one health: best practices to ensure interoperability of genomic data from bacterial pathogens. One Health Outlook. 2020;2:20. 33103064 10.1186/s42522-020-00026-3 PMC7568946

[14] Armstrong GL et al. Pathogen Genomics Public Health. (2019). 10.1056/NEJMsr1813907 PMC7008580 31881145

[15] Washington State Department of. H. One Health.

[16] Page MJ, et al. The PRISMA 2020 statement: an updated guideline for reporting systematic reviews. BMJ. 2021;372:n71. 33782057 10.1136/bmj.n71 PMC8005924

[17] Europe U. N.E.C.f. Generic Statistical Business Process Model. (2009).

[18] WHO. SYSTEMS THINKING for Health Systems Strengthening. (2009).

[19] Ghai RR et al. A generalizable one health framework for the control of zoonotic diseases. Sci Rep 12(2022). 10.1038/s41598-022-12619-1 PMC9124177 35597789

[20] Pelican K, et al. Synergising tools for capacity assessment and one health operationalisation. Rev Sci Tech. 2019;38:71–89. 31564739 10.20506/rst.38.1.2942 PMC8046509

[21] Vesterinen HM, et al. Strengthening multi-sectoral collaboration on critical health issues: One Health Systems Mapping and Analysis Resource Toolkit (OH-SMART) for operationalizing one health. PLoS ONE. 2019;14:e0219197. 31276535 10.1371/journal.pone.0219197 PMC6611682

[22] Benis A, Tamburis O, Chronaki C, Moen A. One Digital Health: a Unified Framework for Future Health ecosystems. J Med Internet Res. 2021;23:e22189–22189. 33492240 10.2196/22189 PMC7886486

[23] Bordier M, et al. Engaging stakeholders in the design of one Health Surveillance systems: a Participatory Approach. Front Vet Sci. 2021;8:646458. 34109232 10.3389/fvets.2021.646458 PMC8180848

[24] Shanbehzadeh M, Nopour R, Kazemi-Arpanahi H. Designing a standardized framework for data integration between zoonotic diseases systems: towards one health surveillance. Inf Med Unlocked 30(2022).

[25] Tripartite OH. Surveillance and Information Sharing Operational Tool. (2022).

[26] Hayman DTS et al. Developing one health surveillance systems. One Health 17(2023). 10.1016/j.onehlt.2023.100617 PMC10665171 38024258

[27] Raymond K, et al. Informatics progress of the Global Burden of Animal diseases programme towards data for one health. Rev Sci Tech. 2023;42:218–29. 37232302 10.20506/rst.42.3365

[28] Neves A et al. The Swiss Pathogen Surveillance platform - towards a nation-wide one health data exchange platform for bacterial, viral and fungal genomics and associated metadata. Microb Genom 9(2023). 10.1099/mgen.0.001001 PMC10272868 37171846

[29] Karimuribo ED, et al. A smartphone app (AfyaData) for innovative one health disease surveillance from community to National Levels in Africa: intervention in Disease Surveillance. JMIR Public Health Surveill. 2017;3:e94. 29254916 10.2196/publichealth.7373 PMC5748470

[30] Uchtmann N, Herrmann JA, Hahn EC, 3rd, Beasley VR. Barriers to, Efforts in, and Optimization of Integrated One Health Surveillance: A Review and Synthesis. *Ecohealth* 12, 368–384 (2015). 10.1007/s10393-015-1022-7 25894955

[31] Mremi IR, Rumisha SF, Sindato C, Kimera SI, Mboera LE. G. comparative assessment of the human and animal health surveillance systems in Tanzania: opportunities for an integrated one health surveillance platform. Glob Public Health, 1–17 (2022). 10.1080/17441692.2022.2110921 35951768

[32] Bordier M, et al. Antibiotic resistance in Vietnam: moving towards a one health surveillance system. BMC Public Health. 2018;18:1136. 30249210 10.1186/s12889-018-6022-4 PMC6154809

[33] Jato-Espino D, Mayor-Vitoria F, Moscardo V, Capra-Ribeiro F, Bartolome Del Pino LE. Toward one health: a spatial indicator system to model the facilitation of the spread of zoonotic diseases. Front Public Health. 2023;11:1215574. 37457260 10.3389/fpubh.2023.1215574 PMC10340543

[34] Pley C, Evans M, Lowe R, Montgomery H, Yacoub S. Digital and technological innovation in vector-borne disease surveillance to predict, detect, and control climate-driven outbreaks. Lancet Planet Health. 2021;5:e739–45. 34627478 10.1016/S2542-5196(21)00141-8

[35] Health TG. A.f.G.a. A federated ecosystem for sharing genomic, clinical data. Science. 2016;352:1278–80. 27284183 10.1126/science.aaf6162

[36] Ope M et al. Regional initiatives in support of surveillance in East Africa: the East Africa Integrated Disease Surveillance Network (EAIDSNet) experience. Emerg Health Threats J 6(2013). 10.3402/ehtj.v6i0.19948 PMC3557906 23362409

[37] Wendt A, Kreienbrock L, Campe A. Zoonotic disease surveillance–inventory of systems integrating human and animal disease information. Zoonoses Public Health. 2015;62:61–74. 24712724 10.1111/zph.12120

[38] Mulder AC et al. HEVnet: a one health, collaborative, interdisciplinary network and sequence data repository for enhanced hepatitis E virus molecular typing, characterisation and epidemiological investigations. Euro Surveill 24(2019). 10.2807/1560-7917.ES.2019.24.10.1800407 PMC6415499 30862334

[39] Pandit N, Vanak AT. Artificial Intelligence and One Health: knowledge bases for causal modeling. J Indian Inst Sci. 2020;100:717–23. 33046950 10.1007/s41745-020-00192-3 PMC7541757

[40] Ecowas. Promoting One Health Approaches through Integrated Data Systems. (2018).

[41] European F, Safety A, et al. Coordinated surveillance system under the One Health approach for cross-border pathogens that threaten the Union - options for sustainable surveillance strategies for priority pathogens. EFSA J. 2023;21:e07882. 36908560 10.2903/j.efsa.2023.7882 PMC9993136

[42] Kaur J, et al. ICMR’s Antimicrobial Resistance Surveillance system (i-AMRSS): a promising tool for global antimicrobial resistance surveillance. JAC Antimicrob Resist. 2021;3:dlab023. 34223098 10.1093/jacamr/dlab023 PMC8210178

[43] Leandro AS, et al. The adoption of the One Health approach to improve surveillance of venomous animal injury, vector-borne and zoonotic diseases in Foz Do Iguacu, Brazil. PLoS Negl Trop Dis. 2021;15:e0009109. 33600424 10.1371/journal.pntd.0009109 PMC7891772

[44] Falzon LC, et al. One health in action: operational aspects of an Integrated Surveillance System for zoonoses in Western Kenya. Front Vet Sci. 2019;6:252. 31417918 10.3389/fvets.2019.00252 PMC6684786

[45] McIntyre KM, et al. A fully Integrated Real-Time detection, diagnosis, and Control of Community Diarrheal Disease Clusters and outbreaks (the INTEGRATE Project): protocol for an enhanced Surveillance System. JMIR Res Protoc. 2019;8:e13941. 31573952 10.2196/13941 PMC6787530

[46] Bracken J. Roadmap to the Digital Transformation of Animal Health Data. Front Vet Sci. 2017;4:123. 28879202 10.3389/fvets.2017.00123 PMC5572109

[47] VanderWaal K, Morrison RB, Neuhauser C, Vilalta C, Perez AM. Translating Big Data into Smart Data for Veterinary Epidemiology. Front Vet Sci. 2017;4:110. 28770216 10.3389/fvets.2017.00110 PMC5511962

[48] Forum WE. Federated Data Systems: Balancing Innovation and Trust in the Use of Sensitive Data. (2019).

[49] Zhang R, et al. From concept to action: a united, holistic and One Health approach to respond to the climate change crisis. Infect Dis Poverty. 2022;11:17. 35144694 10.1186/s40249-022-00941-9 PMC8830086

[50] Tamburis O, Benis A. One Digital Health for more FAIRness. Methods Inf Med. 2022;61:e116–24. 36070786 10.1055/a-1938-0533 PMC9788917

[51] Jin L, et al. Integrating Environmental Dimensions of One Health to Combat Antimicrobial Resistance: essential research needs. Environ Sci Technol. 2022;56:14871–4. 35678702 10.1021/acs.est.2c01651

[52] Gulfidan G, Beklen H, Arga KY. Artificial Intelligence as Accelerator for Genomic Medicine and Planetary Health. Omics (Larchmont N Y). 2021;25:745–9. 10.1089/omi.2021.0170 34780300

[53] Lustgarten JL, Zehnder A, Shipman W, Gancher E, Webb TL. Veterinary informatics: forging the future between veterinary medicine, human medicine, and one health initiatives-a joint paper by the Association for Veterinary Informatics (AVI) and the CTSA One Health Alliance (COHA). JAMIA Open. 2020;3:306–17. 32734172 10.1093/jamiaopen/ooaa005 PMC7382640

[54] K BY et al. Assessing Climate Change Impact on ecosystems and infectious Disease: important roles for genomic sequencing and a one health perspective. Trop Med Infect Dis 5(2020). 10.3390/tropicalmed5020090 PMC7345041 32503239

[55] Gardy JL, Loman NJ. Towards a genomics-informed, real-time, global pathogen surveillance system. Nat Rev Genet. 2018;19:9–20. 29129921 10.1038/nrg.2017.88 PMC7097748

[56] Wendt A, Kreienbrock L, Campe A. Joint use of Disparate Data for the Surveillance of zoonoses: a feasibility study for a one Health Approach in Germany. Zoonoses Public Health. 2016;63:503–14. 26812912 10.1111/zph.12255

[57] Shaikh AT, Ferland L, Hood-Cree R, Shaffer L, McNabb SJ. Disruptive Innovation can prevent the next pandemic. Front Public Health. 2015;3:215. 26442242 10.3389/fpubh.2015.00215 PMC4585064

[58] Peter Rabinowitz MM, Scotch M, PhD MPH, and, Conti L. DVM, MPH. Human and Animal Sentinels for Shared Health risks. Vet Ital. 2009;45:23–4. 20148187 PMC2818012

[59] Duane A. Steward dvm, m., phd, Rosina C. Krecek phd, mba Harry M. Chaddock dvm, eml Julie M. Green dvm, ms Lisa A. Conti dvm, mph. The contribution of biomedical informatics to one health. *JAVMA* 248(2016). 10.2460/javma.248.6.604 26953909

[60] George J, et al. A systematic review on integration mechanisms in human and animal health surveillance systems with a view to addressing global health security threats. One Health Outlook. 2020;2:11. 33829132 10.1186/s42522-020-00017-4 PMC7993536

[61] Mirzaei A, Aslani P, Schneider CR. Healthcare data integration using machine learning: a case study evaluation with health information-seeking behavior databases. Res Social Adm Pharm. 2022;18:4144–9. 35965198 10.1016/j.sapharm.2022.08.001

[62] FAO U. WHO, WOAH. ONE HEALTH JOINT PLAN OF ACTION (2022–2026). (2022).

[63] Zanet S et al. Literature review on worldwide surveillance systems targeting transboundary zoonotic and emerging diseases within the holistic one-health perspective. EFSA Supporting Publications 19(2022).

[64] WHO. Integrated Disease Surveillance and Response Technical Guidelines. (2017).

[65] A GJS. Where the rubber meets the sky: bridging the gap between databases and science. Microsoft Corporation; 2004.

[66] OHEJP. O.a.B. One Health Sequencing for Surveillance HandBook.

[67] Hadfield J, et al. Nextstrain: real-time tracking of pathogen evolution. Bioinformatics. 2018;34:4121–3. 29790939 10.1093/bioinformatics/bty407 PMC6247931

[68] Argimón S et al. Microreact: visualizing and sharing data for genomic epidemiology and phylogeography. Microb Genomics 2(2016). 10.1099/mgen.0.000093 PMC5320705 28348833

[69] Dushoff C, et al. First Nations Health: the need for linked genomic surveillance of SARS-CoV-2. Can Commun Dis Rep. 2022;48:131–131. 35480703 10.14745/ccdr.v48i04a03 PMC9017802

[70] Benis A, Tamburis O. One Digital Health is FAIR. Stud Health Technol Inf. 2021;287:57–8. 10.3233/SHTI210812 34795080

